# NAADP: From Discovery to Mechanism

**DOI:** 10.3389/fimmu.2021.703326

**Published:** 2021-09-07

**Authors:** Timothy F. Walseth, Andreas H. Guse

**Affiliations:** ^1^Department of Pharmacology, University of Minnesota Medical School, Minneapolis, MN, United States; ^2^The Calcium Signalling Group, Department of Biochemistry and Molecular Cell Biology, University Medical Center Hamburg-Eppendorf, Hamburg, Germany

**Keywords:** NAADP, HN1L/JPT2, ryanodine receptor, two-pore channel, calcium signaling

## Abstract

Nicotinic acid adenine dinucleotide 2’-phosphate (NAADP) is a naturally occurring nucleotide that has been shown to be involved in the release of Ca^2+^ from intracellular stores in a wide variety of cell types, tissues and organisms. Current evidence suggests that NAADP may function as a trigger to initiate a Ca^2+^ signal that is then amplified by other Ca^2+^ release mechanisms. A fundamental question that remains unanswered is the identity of the NAADP receptor. Our recent studies have identified HN1L/JPT2 as a high affinity NAADP binding protein that is essential for the modulation of Ca^2+^ channels.

## Introduction

Nicotinic acid adenine dinucleotide phosphate was first described in 1995 as potent Ca^2+^ mobilizing adenine nucleotide (1). NAADP’s biological activity was first observed in sea urchin egg homogenates (1, 2), soon followed by reports describing NAADP evoked Ca^2+^ release in invertebrates (1, 3, 4), mammalian and human cells (5–7). Obviously, NAADP signaling was very successful in evolution since it operates in both invertebrate and vertebrates.

Here, we will highlight the steps of NAADP’s discovery, the tales and mysteries of its mode of action, and finally discuss the unifying hypothesis of NAADP action that was published some years ago (8).

## NAADP: Discovery in Sea Urchin Egg Homogenate

Hon Cheung Lee’s laboratory at the University of Minnesota developed the sea urchin egg homogenate system in 1985 in order to study d-*myo*-inositol 1,4,5-trisphosphate (IP_3_) induced Ca^2+^ mobilization *in vitro* (2). The egg homogenate system contained Ca^2+^ pumps that were able to pump Ca^2+^ into vesicles in an ATP-dependent manner and IP_3_ receptors that would release Ca^2+^ from the vesicles in response to the addition of IP_3_. Two important observations using the sea urchin egg homogenate system were made in 1987 (9). The first was that nicotinamide adenine dinucleotide (NAD) was able to release Ca^2+^, but only after a lag of several minutes. This observation led to the discovery of cyclic ADP-ribose (cADPR) (10). The lag in the response to NAD was due to the conversion of NAD to cADPR by ADP-ribosyl cyclases. The second observation was that nicotinamide adenine dinucleotide (NADP) caused an immediate and robust release of Ca^2+^ from the egg homogenate (9). Further examination of the NADP-induced Ca^2+^ release revealed that the release was not due to NADP itself, but a contaminant in commercial sources of NADP that could be resolved chromatographically from NADP (1). Alkaline treatment of NADP produced a 30-fold enrichment of the active metabolite that released Ca^2+^ (1). In 1995, Aarhus and Lee demonstrated that the derivative produced by alkaline treatment of NADP was nicotinic acid adenine dinucleotide 2’-phosphate (NAADP) (1). Some of the basic properties of NAADP-induced Ca^2+^ release as determined using the sea urchin egg system are as follows. Ca^2+^ release by NAADP was saturable with an EC_50_ of 30nM, making it the most potent of the agents (IP_3_ and cADPR) known to mobilize Ca^2+^ from intracellular stores (1). NAADP-induced Ca^2+^ release exhibited self-desensitization, but the action of NAADP was not desensitized by preexposure to IP_3_ or cADPR (1). Antagonists of IP_3_ or cADPR did not block NAADP-induced Ca^2+^ release (1). In addition, the Ca^2+^ stores sensitive to NAADP were distinct than those released by IP_3_ or cADPR as indicated by density gradient fractionation of egg homogenates and stratifying live sea urchin eggs by centrifugation. While IP_3_ and cADPR appear to release Ca^2+^ from endoplasmic reticulum stores, NAADP releases Ca^2+^ from a thapsigargin-insensitive store that has the properties consistent with being acidic lysosomal type organelles (11, 12). In the sea urchin egg system, NAADP displays a unique self-inactivation mechanism (13, 14). Subthreshold concentrations of NAADP inhibit subsequent Ca^2+^ release by maximal concentrations of NAADP in a time and concentration-dependent manner (13, 14). NAADP was shown to be active in intact cells as microinjection of NAADP into live sea urchin eggs resulted in Ca^2+^ mobilization and induced a cortical reaction (1). NAADP has now been shown to be active in many cell types [reviewed in references (15–18)].

## NAADP: Endo/Sarcoplasmic Reticulum and Ryanodine Receptors

The molecular mechanisms involved in Ca^2+^ mobilizing activity of NAADP have been a matter of many discussions in the past years: basically, two hypotheses evolved: (i) NAADP activates type 1 ryanodine receptor (RYR1) localized on endoplasmic reticulum (ER) Ca^2+^ stores, or (ii) NAADP’s target organelles are acidic endo-lysosomal stores and Ca^2+^ mobilization proceeds *via* two-pore channels (TPC).

In 1999 Cancela et al. proposed a model for pancreatic acinar cells in which nanomolar concentrations of NAADP activate an unknown NAADP receptor/Ca^2+^ channel that releases trigger Ca^2+^ which then would be amplified by Ca^2+^ induced Ca^2+^ release (CICR) through RYR (19). A more direct effect of NAADP on cardiac RYR (RYR2) was reported in 2001 showing Ca^2+^ release from cardiac microsomes; further, RYR2s in lipid planar bilayers were activated by NAADP (20). However, micromolar NAADP was used in (20) while in most other studies nanomolar NAADP was sufficient to evoke Ca^2+^ release. One year later, in a similar experimental approach in lipid planar bilayers, low nanomolar NAADP (EC_50_ ~ 30 nM) increased the open probability of RYR1 from skeletal muscle of RYR1 (21). However, others did not confirm RYR activation by NAADP in lipid planar bilayers (22, 23). In 2003, in the nuclear envelope of pancreatic acinar cells, NAADP-evoked Ca^2+^ release was not affected by inhibition of lysosomal acidification, but was blocked by antagonists of RYR, ryanodine and ruthenium red, as well as by depletion of ER using SERCA inhibitor thapsigargin; accordingly, it was hypothesized that NAADP acts on RYR, but most likely indirectly *via* a NAADP binding protein (NAADP BP) (24). In 2004 and 2005, RYR was identified as major Ca^2+^ channel responding to NAADP in T cells; using a combination of NAADP microinjection during Ca^2+^ imaging, NAADP evoked local and global Ca^2+^ signaling was abolished by either pharmacological inhibition or gene silencing of RYR (25). More evidence for NAADP acting on RYR1 was obtained in partially purified RYR1 preparations where NAADP facilitated [^3^H]ryanodine binding, while this was blocked by a novel NAADP antagonist, BZ194 (26). Collectively, these data indicated that if an unknown NAADP receptor/Ca^2+^ channel, as proposed by Cancela et al. (19), would be involved to produce trigger Ca^2+^ ahead of activation of RYR by CICR, this process must be working with very small and very fast Ca^2+^ signals. To solve this problem, at least partially, high-resolution Ca^2+^ imaging was optimized for T cells and combined with NAADP microinjections and specific gene knock-outs (27). Using high spatiotemporal resolution (25 ms, 368 nm), for the first time initial local Ca^2+^ signals of T cells were characterized; while experiments with *Ryr1^-/-^, Orai1^-/-^, Stim1^-/-^* and *Stim2^-/-^* T cells identified the protein products of these genes as major elements essential for NAADP evoked Ca^2+^ signaling, evidence for involvement of other ion channels was not obtained (27, 28). Though further studies from Petersen and colleagues confirmed that NAADP acts on ER stores *via* RYR in pancreatic acinar cell function, they also emphasized that acidic stores and two-pore channels (TPC) are additionally required (29).

However, as mentioned above, in a couple of systems, RYR1 did not respond to NAADP. Using overexpression of RYR1 in HEK cells and intracellular dialysis of 10 nM NAADP did not evoke Ca^2+^ signals above background (30). In a similar cell model, HEK cell overexpressing RYR1, direct activation NAADP (30 nM and 1 µM) activation of RYR1 by on-nucleus patch clamp was not observed (31).

Taken together, these studies suggest two different roles for RYR in NAADP signaling: (i) as amplifier of initial lysosomal Ca^2+^ signals, or (ii) in a more direct sense as NAADP sensitive Ca^2+^ channels. However, the latter results appear to be restricted to T cells and pancreatic acinar cells. While in pancreatic acinar cells also the endo-lysosomal system and TPCs were found to be involved in NAADP signaling, in T cells a role of endo-lysosomes remains to be confirmed (32).

## NAADP: Acidic Stores and Two-Pore-Channels

The second hypothesis regarding NAADP’s mechanism of action is that NAADP targets organelles that are acidic endo-lysosomal stores and Ca^2+^ mobilization proceeds *via* two-pore channels (TPCs) Several laboratories have demonstrated that the two-pore channel (TPC) family of endolysosomal proteins are regulated by NAADP (33–35). The experimental approaches utilized to support the role of TPCs in NAADP signaling, include manipulation of TPC levels by overexpression (33–36) or knockdown (33, 34), as well as electrophysiological analyses (36–39). However, in some reports TPCs were found to be activated primarily by phosphatidylinositol 3,5-bisphosphate and to conduct Na^+^ currents rather than Ca^2+^ currents (40–42).

An important unresolved issue is whether TPCs directly interact with NAADP. Most biological data suggest that while TPCs are required for NAADP action, NAADP does not appear to bind directly to TPCs. For instance, overexpression of mammalian TPC2 slightly increased [^32^P]NAADP binding activity, but the increment in binding was much lower than the increase in TPC2 mRNA levels (3-fold *versus* 250-fold) (34). [^32^P]NAADP binding activity was also found in immunoprecipitates using antibodies to sea urchin TPCs (39). The question of whether NAADP binds directly to TPCs was assessed by photoaffinity labeling. [^32^P]-5-azido-NAADP was synthesized and characterized as a photoaffinity probe for NAADP binding sites (43–46). 5-azido-NAADP was previously shown to release Ca^2+^ from sea urchin egg homogenates and mammalian cells with high affinity (44, 47). Photoaffinity labeling of sea urchin egg homogenates with [^32^P]-5-azido-NAADP resulted in specific labeling of proteins with molecular weights of 45, 40 and 30kDa, which are much smaller in size than the TPCs expressed in sea urchin (45). These proteins exhibited the properties of high affinity [^32^P]-NAADP binding previously described in this system (45). The photolabeled proteins were not recognized by antibodies to sea urchin TPCs suggesting these proteins are immunologically distinct from TPCs (45). A small amount (~5%) of the photolabeled 45 and 40kDa proteins were pulled down with antibodies to sea urchin TPCs, suggesting an interaction between these proteins and TPCs (45).

Photolabeling in mammalian cell extracts with [^32^P]-5-azido-NAADP resulted in the specific labeling of 23kDa protein(s) (44, 46). The photolabeling pattern was not changed by overexpression of TPC isoforms (44). Photolabeling was also unchanged in tissue samples form TPC1 or TPC2 knockout mice (44). Similar results were also obtained with a TPC1/TPC2 double knockout mouse model (48). The unchanged photolabeling from overexpression and knockout models indicates that the high affinity NAADP binding proteins detected by [^32^P]-5-azido-NAADP in mammalian systems are independent of TPC proteins. Overall, the data suggest that a NAADP-sensitive complex containing the TPC channel and high affinity NAADP binding protein(s) is responsible for mediating NAADP-evoked Ca^2+^ release.

The identity of the high affinity NAADP binding proteins identified *via* photoaffinity labeling is crucial to our understanding of the mechanism by which NAADP elicits Ca^2+^ release. The next section details our efforts to accomplish this task.

## NAADP: Unifying Hypothesis

One excellent possibility to identify proteins that specifically bind small ligands is photoaffinity labelling. In 2012 independent studies reported photoaffinity labelling experiments with [^32^P]-5-N_3_-NAADP as PAL-ligand (44–46). The main, but unexpected finding of all three reports was labelling of small soluble proteins in mammalian cell extracts (44, 46). In the two reports conducted in different mammalian cell lines and tissues, a protein double band of 22/23kDa was labelled consistently by [^32^P]-5-N_3_-NAADP and specificity was demonstrated by displacement of the label by low nanomolar concentrations of ‘cold’ NAADP (44, 46).

These novel findings resulted in the ‘unifying hypothesis’ (8). The central idea of this hypothesis consists of a (small) NAADP binding protein and a Ca^2+^ channel that is activated by the NAADP binding protein in conjunction with NAADP. This idea builds on an earlier report by Petersen’s group where binding proteins for NAADP or cADPR were proposed to activate RYR (24). The ‘unifying hypothesis’ helps to explain the fact that one part of the community obtained evidence of RYR1 localized on the ER as target of NAADP, but not for lysosomal TPCs, while the other part reported data supporting TPCs localized on lysosomes as NAADP’s target channels, but did not find evidence for RYR in NAADP signaling (for details see chapters above).

**Figure 1:**
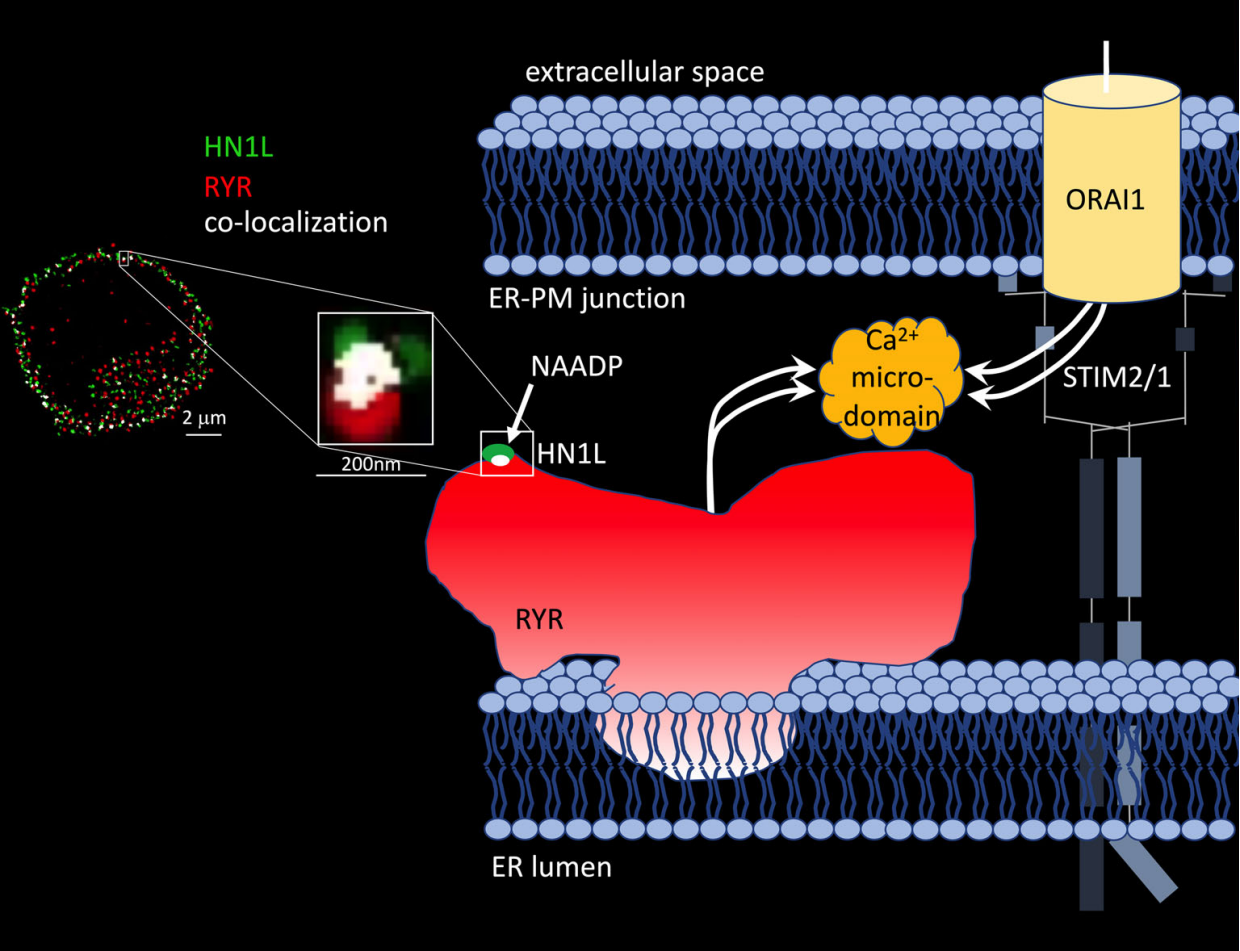
HN1L/JPT2 co-localization with ryanodine receptors and scheme of Ca^2+^ microdomain formation by ryanodine receptors and ORAI1 channels in ER-PM junctions of T cells. Left: Co-localization of HN1L/JPT2 with RYR shown by super-resolution microscopy in a single Jurkat T cell [image taken from Figure 6 of (45)]. From (52). Reprinted with permission from AAAS. Right: scheme of Ca^2+^ microdomain formation by RYRs and ORAI1 channels in ER-PM junctions of T cells. Abbreviations used: STIM1/2, stromal interaction molecule 1 and/or 2.

The molecular identification of the protein hidden in the 22/23kDa band that was photoaffinity labelled turned out to be as complicated as the design of NAADP analogues suitable for photo-affinity labelling (48–50). Nine years after discovery of the small soluble NAADP binding proteins and formulation of the ‘unifying hypothesis’, two back-to-back studies in *Science Signaling* reported identification of a 21kDa NAADP binding protein as haematological and neurological expressed 1-like protein (HN1L)/Jupiter microtubule associated homolog 2 (JPT2) in March 2021 (51, 52). HN1L/JPT2 was purified independently from erythrocytes and Jurkat T-lymphocytes using photo-affinity labeling as selection criterion during protein purification and enrichment steps. HN1L/JPT2, also known as C16orf34, FLJ13092, KIAA1426, or L11, is a 20.1 kDa protein with broad expression in mammalian cell types (see human protein atlas.org) and with orthologues throughout the animal kingdom (51). Recombinantly expressed HN1L/JPT2 was specifically photo-affinity labelled, though displacement by ‘cold’ NAADP was somewhat shifted to higher NAADP concentrations (51, 52). Crucial experiments to validate HN1L/JPT2’s role as signal transducer in NAADP signaling were (i) decreased responsiveness of SKBR cells to microinjected NAADP upon gene silencing of HN1L/JPT2 by shRNA (44) and (ii) largely diminished initial Ca^2+^ microdomains upon knock out of *Hn1l/Jpt2* using CRISPR/Cas technology both in human Jurkat T cells or in primary rat effector T cells (51). Further, in *Hn1l/Jpt2^-/-^* rat T cells, NAADP antagonist BZ194 did not further enhance the Ca^2+^ phenotype suggesting that the same signaling pathway is affected by both interventions (52). These results confirmed the first part of the ‘unifying hypothesis’ since the NAADP binding protein is not any more a faint band on a phosphoscreen, but an identified protein to be further analysed and validated by the scientific community. But what about the second part of the ‘unifying hypothesis’, activation of different ion channels? Roggenkamp et al. (52) reported co-localization of HN1L/JPT2 with RYR already before T cell receptor (TCR)/CD3 stimulation using super-resolution microscopy (**Figure 1**) (52). Further, TCR/CD3-dependent re-localization of HN1L/JPT2 from the cytosol towards the plasma membrane within seconds was observed by super-resolution microscopy. Finally, HN1L/JPT2 was detected by western blot in anti-RYR immunoprecipitates from Jurkat T cells (52). Collectively these data confirm interaction of HN1L/JPT2 with RYR.

**Figure 2:**
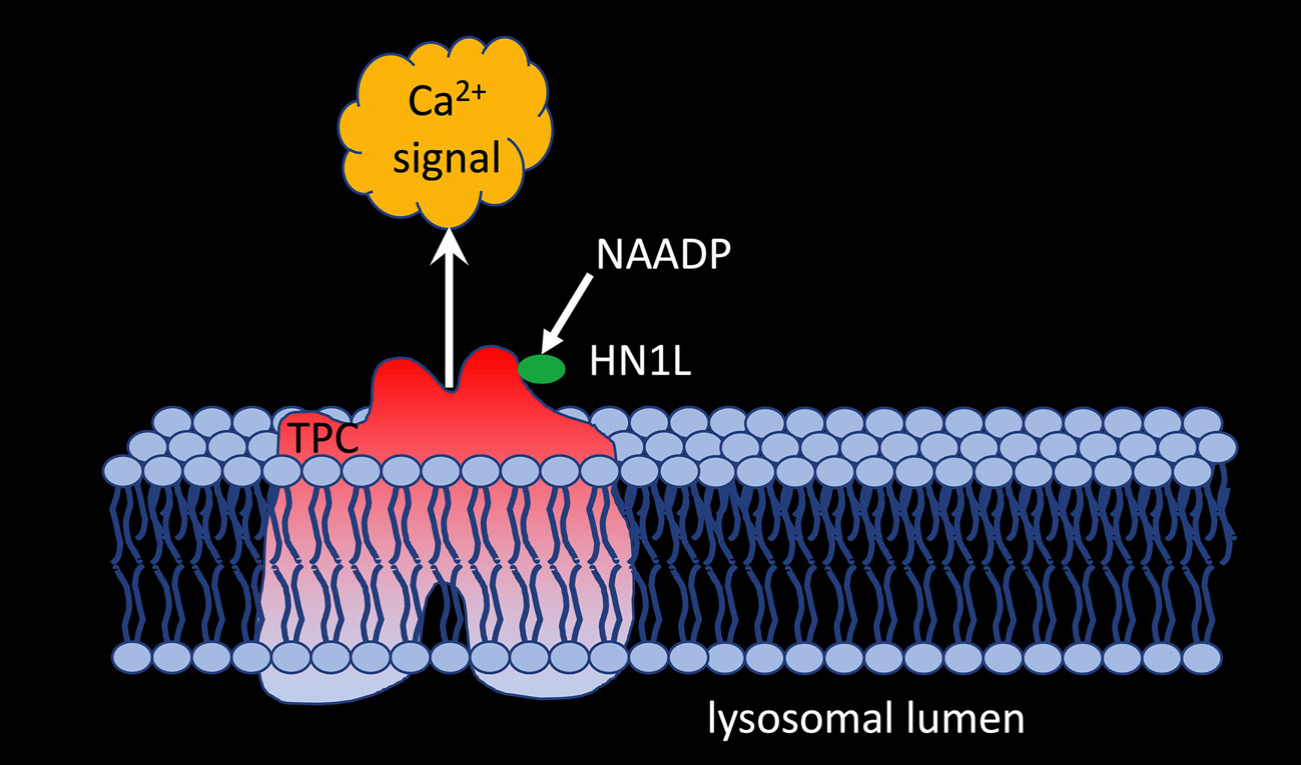
HNL1/JPT2 interacts with TPC1 and is essential for NAADP-evoked calcium signaling *via* two pore channels.

But what about other proposed target channels for NAADP? Gunaratne et al. (51) demonstrated co-immunoprecipitation of HN1L/JPT2 when TPC1, but not TPC2, was pulled down from HEK293 cells overexpressing GFP-tagged TPC1 or TPC2 demonstrating that HN1L/JPT2 may function as a switch point to direct incoming signals either to RYR1 or TPC1 activation (**Figure 2**). The fact that HN1L/JPT2 interacts with two different ion channels nicely confirms the second part of the ‘unifying hypothesis’ and paves the way for experiments to unravel the molecular mechanism(s) opening this bifurcation onto one or the other path.

## Discussion and Outlook

Despite confirmation of the main aspects of the ‘unifying’ hypothesis, several questions remain open. Though photoaffinity labelling of HN1L/JPT2 and specific and high affinity binding of [^32^P]NAADP were demonstrated (51, 52), details of the molecular basis for NAADP binding to HN1L/JPT2 remain elusive. Under non-reducing conditions, recombinant HN1L/JPT2 was detected in size exclusion-HPLC mainly as dimer, though higher oligomers and monomers were observed, too (52). It is currently unclear whether this might be an artefact due to recombinant HN1L/JPT2 production, or whether there is a biological background, perhaps related to regulation of HN1L/JPT’s activity in NAADP signaling. Further, the binding site of NAADP on HN1L/JPT2 so far has not been mapped. However, understanding binding of NAADP would greatly facilitate further structure-activity studies to translate the basic finding of a novel signal transducer to pharmacology and hopefully to therapy. What type of disease and therapy is meant? In 2010 it was shown that NAADP signaling is an important determinant of CNS autoimmunity (53); however, specific, high affinity compounds antagonizing NAADP’s interaction with HNL1 that may be tested in multiple sclerosis animal model experimental autoimmune encephalomyelitis are not yet known.

Next open question relates to the molecular basis for HN1L/JPT2 binding to and activation of different Ca^2+^ channels. Like for NAADP binding, we do not know the binding interface of either side. However, also this protein-protein interaction would be of high translational value for the same reasons as quoted for the NAADP binding site at HN1L/JPT2 above. Another point of interest is the interaction of HN1L/JPT2 with TPC isoforms. Co-immunoprecipitation was only confirmed for TPC1, but not for TPC2 (51). However, the molecular basis for this is unclear. This differential binding of HN1L/JPT2 to TPC1, but not TPC2, also opens the question of further, still unknown members of the NAADP signalosome.

Further, the exact kinetic mechanism of NAADP-HN1L/JPT2-Ca^2+^ channel interaction is unknown. Possible would be (i) that NAADP binds HN1L/JPT2 first and afterwards to the Ca^2+^ channel or (ii) that a fraction of HN1L/JPT2 is already bound to the Ca^2+^ channel before cell activation and operates like in a “waiting position” until NAADP formation upon cell activation takes place.

It is possible that other NAADP binding proteins exist and participate in NAADP signalsome. Zhang et al. have identified Lsm12 as a high affinity NAADP binding protein that is essential for NAADP -induced TPC2 activation (54). The Lsm12 data suggest that NAADP signaling has more components that need to be identified and studied.

In conclusion, the identification of HN1L/JPT2 constitutes a milestone in Ca^2+^ signaling research, allowing for future characterization of the molecular basis as well as for translational research towards therapeutic applications. The ‘unifying hypothesis’ was confirmed in its major aspects, however, the current model does not explain the complexity and calls for further investigation of the NAADP signaling pathway.

## Data Availability Statement

The original contributions presented in the study are included in the article/supplementary material. Further inquiries can be directed to the corresponding author.

## Author Contributions

Both authors wrote the manuscript. All authors contributed to the article and approved the submitted version.

## Funding

This work was supported by the Deutsche Forschungsgemeinschaft (DFG) (Project number 335447717; SFB1328, project A01 to AG). Research in the Guse lab is also supported by the Joachim-Herz-Foundation (Hamburg; Infectophysics consortium, project 4), NCL-Foundation (Hamburg), and EU project INTEGRATA - DLV-813284. Research in the Walseth lab is supported by NIH R15-GM131129.

## Conflict of Interest

The authors declare that the research was conducted in the absence of any commercial or financial relationships that could be construed as a potential conflict of interest.

## Publisher’s Note

All claims expressed in this article are solely those of the authors and do not necessarily represent those of their affiliated organizations, or those of the publisher, the editors and the reviewers. Any product that may be evaluated in this article, or claim that may be made by its manufacturer, is not guaranteed or endorsed by the publisher.
